# Pre-culture Sudan Black B treatment suppresses autofluorescence signals emitted from polymer tissue scaffolds

**DOI:** 10.1038/s41598-017-08723-2

**Published:** 2017-08-21

**Authors:** Lin Qi, Erin K. Knapton, Xu Zhang, Tongwen Zhang, Chen Gu, Yi Zhao

**Affiliations:** 1 Laboratory for Biomedical Microsystems, Department of Biomedical Engineering, The Ohio State University, Columbus, Ohio 43210 USA; 2 Department of Biological Chemistry and Pharmacology, The Ohio State University, Columbus, Ohio 43210 USA

## Abstract

In tissue engineering, autofluorescence of polymer scaffolds often lowers the image contrast, making it difficult to examine cells and subcellular structures. Treating the scaffold materials with Sudan Black B (SBB) after cell fixation can effectively suppress autofluorescence, but this approach is not conducive to live cell imaging. Post-culture SBB treatment also disrupts intracellular structures and leads to reduced fluorescence intensity of the targets of interest. In this study, we introduce pre-culture SBB treatment to suppress autofluorescence, where SBB is applied to polymeric scaffold materials before cell seeding. The results show that the autofluorescence signals emitted from polycaprolactone (PCL) scaffolds in three commonly used fluorescence channels effectively decrease without diminishing the fluorescence signals emitted from the cells. The pre-culture SBB treatment does not significantly affect cell viability. The autofluorescence suppressive effect does not substantially diminish during the culturing period up to 28 days. The results also show that cell migration, proliferation, and myogenic differentiation in pre-culture SBB-treated groups do not exhibit statistical difference from the non-treated groups. As such, this approach greatly improves the fluorescence image quality for examining live cell behaviors and dynamics while the cells are cultured within autofluorescent polymer scaffolds.

## Introduction

Polymeric biomaterials are widely used as cell culturing scaffolds in tissue engineering. Cell interactions with the scaffolds are critical for cell proliferation, differentiation, and tissue growth^1–3^. A variety of engineering approaches has been developed to examine and quantify cell responses, including scanning electron microscopy (SEM), flow cytometry, western blotting, and fluorescence labeling^4–6^. Among these, flow cytometry and western blotting can quantitatively determine the expressions of proteins in cells. Unfortunately, these approaches cannot reveal the morphology relationship between cells and scaffolds, making it difficult to study cell responses to specific stimulations applied by the scaffolds or the environment. SEM provides a powerful tool to unveil cell morphological responses in three-dimensional formats. The examination of protein expressions, however, is difficult. This has been addressed by using immunogold SEM, where gold nanoparticles are used to immunolabel the targets of interest before the samples are prepared for SEM observation^7^. Nonetheless, SEM requires a fairly complicated and lengthy sample preparation process. The fixed cells are often vulnerable to cracking and distortion. In addition, SEM is only applicable to end-point examinations. *In-situ* observation of live cell responses is difficult.

In view of these limitations, fluorescence labeling is used as a primary approach to investigate the interactions between cells and polymeric scaffolds. The fluorescence dyes are bounded to corresponding molecules in the cells through chemical reactions or physical adsorption. This allows for the investigation of cell responses to scaffolds and environment (*e*.*g*. cell morphology change, protein expression, and distribution) in live and fixed cells without disrupting cell-scaffold morphology relationship. Nonetheless, the efficacy of fluorescence labeling is limited by autofluorescence of scaffold materials, which emit fluorescence signals that are difficult to be distinguished from the signals emitted from externally added fluorescence markers. In addition, some materials that have been added into the scaffolds to modify the physical properties (*e*.*g*. electric conductivity, mechanical properties, etc.) also have autofluorescence signals^8, 9^. The image quality is thus affected.

The autofluorescence interference can be addressed by staining the targets of interest with a labeling dye that has the excitation wavelength outside the excitation spectrum of autofluorescent scaffold materials^10^. Such approaches, however, often require the use of near infrared fluorescent dyes. This is not compatible with the common filters in fluorescence microscopy. Another approach to reducing autofluorescence is by photobleaching the polymer scaffolds prior to cell culturing. The fluorophores in the scaffolds are consumed through UV induced or laser induced chemical interactions with molecular oxygen^11^. In this approach, autofluorescence signals of the irradiated polymers cannot be fully eliminated and may recover up to 20% after one hour of treatment^12^. The autofluorescence interference can also be reduced using digital image processing^13^. This is based on the fact that the fluorescence probes for subcellular structures often have narrow excitation/emission ranges, while the autofluorescence signal has a fairly wide range^14–16^. The fluorescence image contrast can thus be enhanced by subtracting the background signals at a different wavelength. The method is however highly affected by the variations of autofluorescence signal at different wavelengths. It is also not applicable to the cases with low fluorescence intensities^17^.

**Figure 1:**
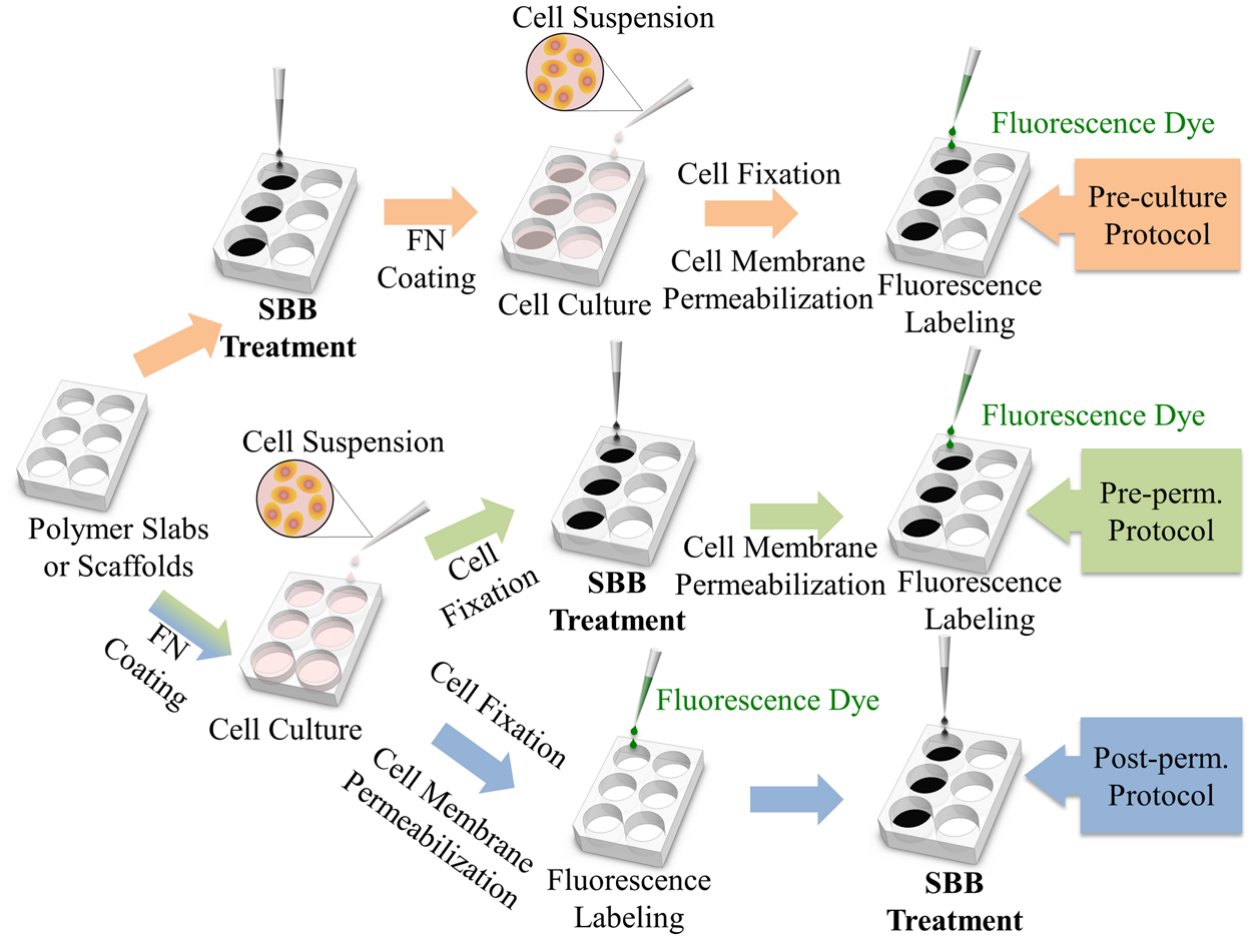
Three SBB treatment schemes. In pre-culture SBB treatment, the scaffold is treated with SBB prior to cell culturing. In pre-permeabilization (pre-perm.) SBB treatment, the scaffold is treated right after cell fixation, and followed by cell membrane permeabilization and the remaining steps of fluorescence labeling. In post-permeabilization (post-perm.) approach, the scaffold is treated after fluorescence labeling.

In addition to the above approaches, Sudan Black B (SBB) has been widely used in neurological histology to decrease the autofluorescence of lipofuscin in brain tissues and to study a variety of lipid-based compounds^18–20^. In recent studies, SBB has been proven its capability to reduce undesired background fluorescence emitted from multiple types of polymeric scaffold materials, including poly(l-lactide–co-ε-caprolactone) (PLCL), poly(lactic-co-glycolic acid) (PLGA), poly(urethane) (PU)^21^, and silk^22^. Jaafar *et al*. claimed that the autofluorescence quenching effect of SBB is due to its large light absorption capability and the smoothing effect^21^. In current practices, SBB treatment is performed after cell fixation (either before or after cell membrane permeabilization as illustrated in Fig. 1). This is because Sudan dyes with azo structures are considered genotoxic and carcinogenic^23, 24^. Although the cytotoxicity of SBB has not yet been well studied, it is hazardous for skin and toxic for mucus membranes^25^. SBB based autofluorescence suppression has yet been used for live cell imaging. This limits the wide adoption of SBB treatments in investigating many dynamic cellular and molecular processes, such as Ca^2+^ flux, cell migration, and real-time cell responses upon electric and/or mechanical stimulations, etc.^26–28^.

In this study, we extended the use of SBB based autofluorescence suppression to live cell imaging. The SBB treatment was performed right after polymeric scaffolds preparation and before cell seeding (Fig. 1). The capability of autofluorescence suppression was evaluated by culturing cells on polycaprolactone (PCL) scaffolds, and examining cell viability and behaviors using four cell lines: fibroblasts, endothelial cells, skeletal myoblasts, and endodermal cells, as well as primary cells from rat hippocampi. The endurance of SBB autofluorescence suppression was examined during the culturing period up to 28 days. The results showed that pre-culture SBB treatment can effectively suppress the autofluorescence of polymeric scaffold materials without significantly changing cell growth and responses.

## Results

### The optimal SBB concentration

**Figure 2:**
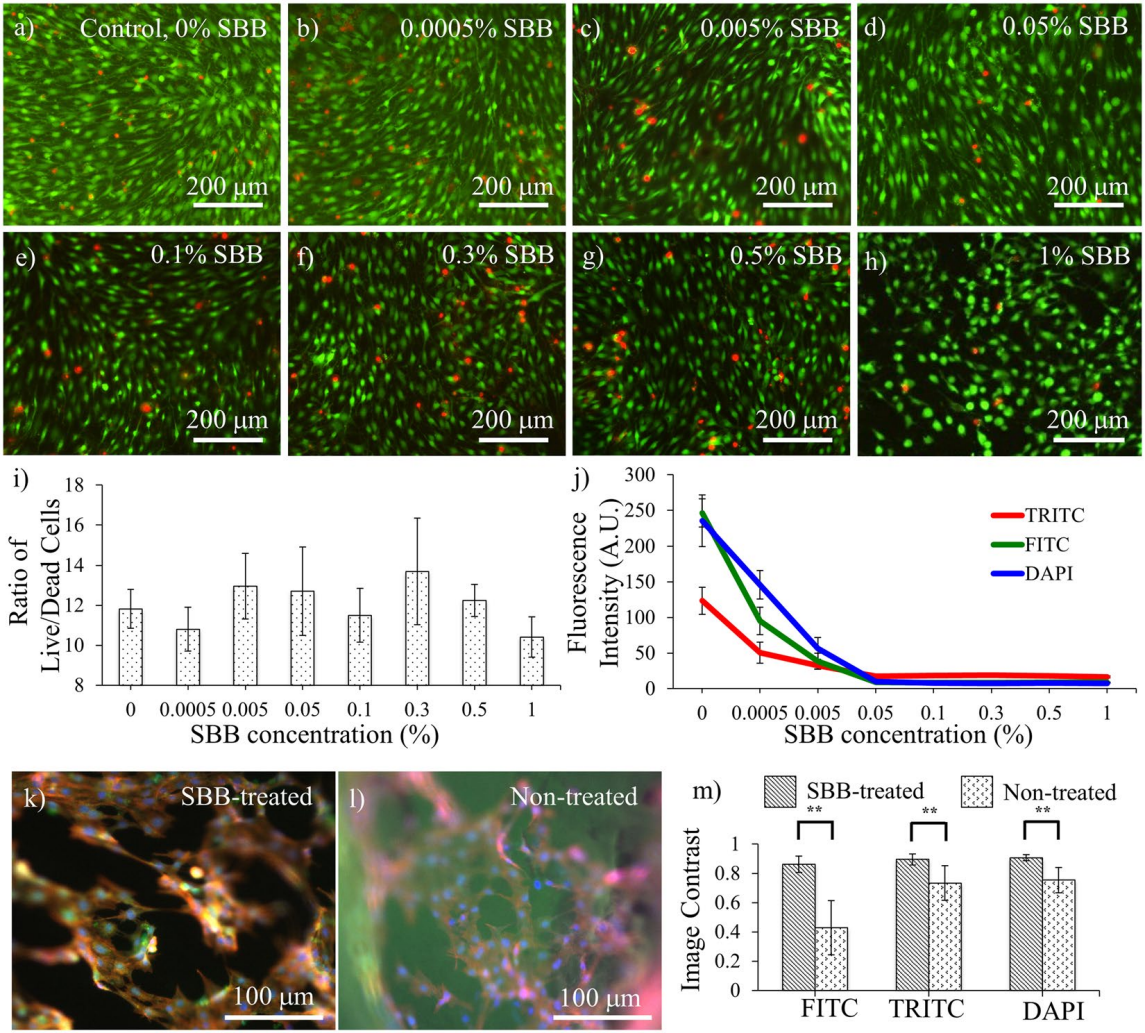
The changes of cell viability and autofluorescence suppression with the SBB concentration. (**a**–**h**) Fluorescence images showing live and dead cells of 3T3 fibroblasts on PCL slabs with different SBB concentrations. Scale bar = 200 μm. (**i**) The change of the ratio of live/dead cells with the SBB concentration. No significant difference was found among the groups with different SBB concentrations (p > 0.05). (**j**) The fluorescence intensity of the autofluorescence of PCL slabs treated with 0% to 1% SBB concentrations. (**k** and **l**) The representative fluorescence images of the PCL slabs cultured with 3T3 fibroblasts: (**k**) with 0.3% SBB and (**l**) non-treated. (**m**) The image contrasts of the fluorescence images of the PCL slabs cultured with 3T3 fibroblasts. For (**a**)–(**h**), the cells were stained by live/dead assay on Day 3 of cell culturing. Green denotes live cells; red denotes dead cells. For (**k**) and (**l**), the cells were stained by calcein AM, rhodamine-phalloidin, and DAPI on Day 3. Green denotes cytoplasm; red denotes F-actins; and blue denotes cell nuclei.

The SBB concentration should be adjusted to the value that can suppress the autofluorescence of polymer scaffolds while not substantially affecting cell viability. An experiment was performed to determine the appropriate SBB concentration (Fig. 2a–h). 3T3 fibroblasts were used ﻿a﻿nd the culture lasted for 3 days before examination. The results showed the average live/dead cells ratio reached its maximum at 0.3% SBB concentration. The ratio dropped as the SBB concentration kept increasing (Fig. 2i). The image contrast increased with the increasing SBB concentration when the SBB concentration was below 0.1% (Fig. 2a–d). There was no statistical difference in the image contrast when the SBB concentration was above 0.1%. The cells on 1% SBB treated PCL slabs had relatively lower confluence than other groups. Some cells in this group showed rounded shapes (Fig. 2h). This phenomenon was not observed in the groups with lower SBB concentrations.

The capability of autofluorescence suppression of SBB treatment was represented by the fluorescence signal reduction compared to the non-treated PCL groups. The results showed that the autofluorescence of the SBB-treated PCL slabs decreased with the increasing SBB concentration in all the three fluorescence channels (TRITC, FITC, and DAPI) (Fig. 2j). The autofluorescence suppressive efficacy reached the maximum at 0.05% SBB in FITC and TRITC channels, and at 0.1% in DAPI channel. When the SBB concentration kept increasing, there were no statistical changes in the fluorescence intensity: p = 0.2438 between 0.05% and 0.1% groups in TRITC channel; p = 0.6795 between 0.05% and 0.1% groups in FITC channel; and p = 0.4157 between 0.1% and 0.3% groups in DAPI channel. Therefore, the SBB concentration >0.1% is sufficient to maintain the maximal autofluorescence suppressive effect for all the three fluorescence channels.

Considering both the cell viability and autofluorescence suppressive efficacy, the SBB concentration of 0.3% is chosen in this study. Figure 2k provides a representative fluorescence image of a cell-cultured PCL slab with 0.3% SBB treatment. After 3 days of cell culturing, the SBB treatment effectively suppressed autofluorescence signals emitted from PCL, leading to clear boundaries of subcellular structures. The image contrast sharply increased comparing to the control groups without SBB treatments (Fig. 2l). The differences in the image contrast between the SBB-treated slabs and the corresponding controls were highly statistically significant for all the three fluorescence channels (p < 0.0001, p = 0.0024, and p = 0.0003 for FITC, TRITC, and DAPI channels, respectively) (Fig. 2m). Among these, FITC channel exhibited the greatest difference in the image contrast. The average image contrast of the SBB-treated PCL slabs (0.862 ± 0.057) was more than two times of the control group (0.429 ± 0.185).

### Cell viability tests

**Figure 3:**
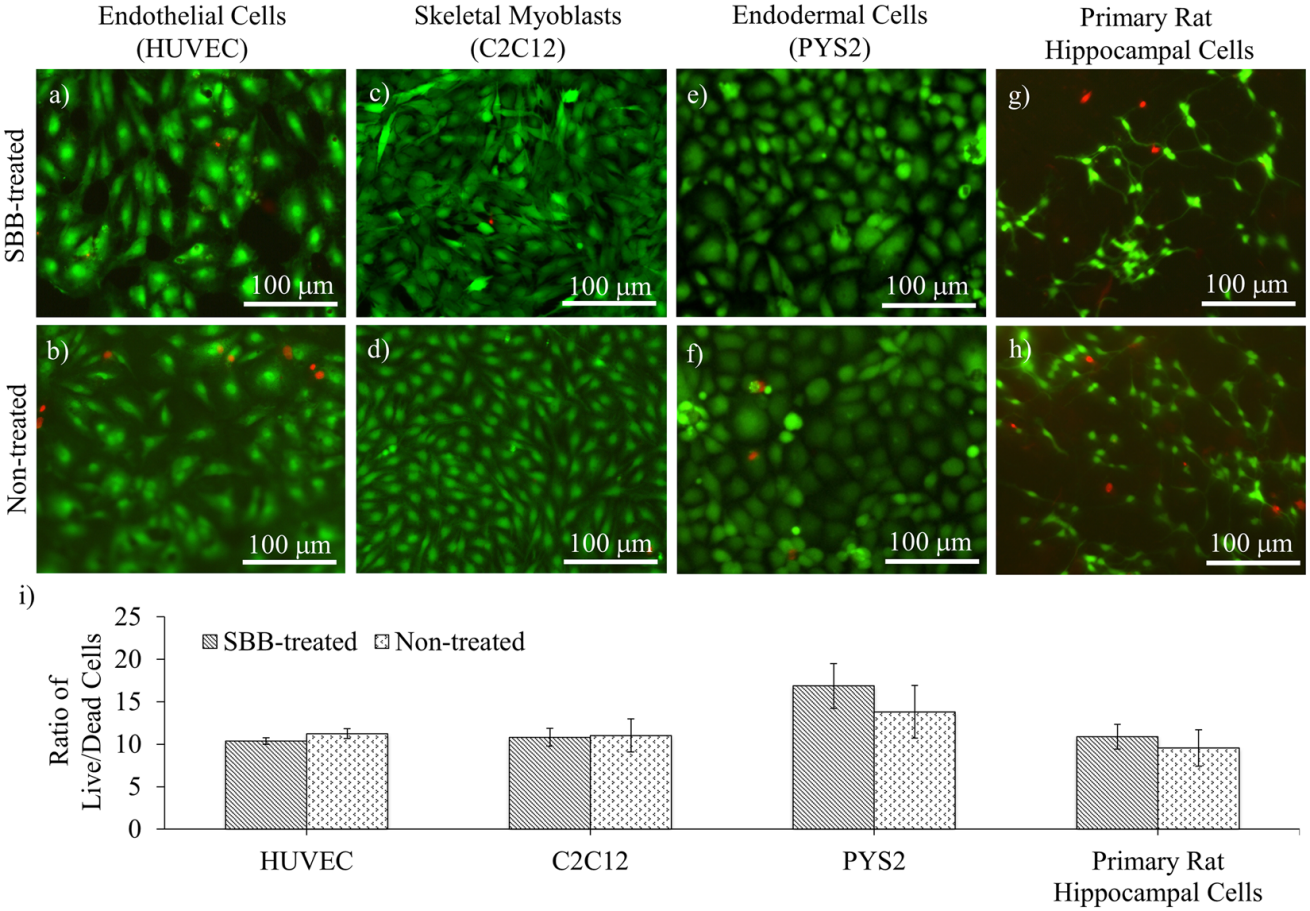
The effect of SBB treatment on live/dead cells ratio. Cell viability of three cell lines, (**a** and **b**) endothelial cells (HUVEC), (**c** and **d**) skeletal myoblasts (C2C12), (**e** and **f**) endodermal cells (PYS2), and (**g** and **h**) primary cells from rat hippocampi on 0.3% SBB-treated and non-treated PCL slabs were examined by calculating the ratios of live/dead cells (**i**). The cells were stained by live/dead assay on Day 3 of cell culturing. In particular, primary hippocampal neurons can be identified by their neuronal processes. Live cells are labeled in green. Dead cells are labeled in red.

To investigate whether the SBB treatment with the selected concentration (0.3%) affects cell viability, live/dead assays were performed using human vein umbilical endothelial cells (HUVECs, Fig. 3a and b), skeletal myoblasts (C2C12, Fig. 3c and d), endodermal cells (PYS2, Fig. 3e and f) and primary cells from rat hippocampi (Fig. 3g and h) after culturing the cells on SBB-treated PCL slabs for 3 days. The results showed that there was no statistical difference in the ratios of live/dead cells between the SBB-treated and non-treated groups (p = 0.0953, 0.8505, 0.1851, 0.5524 for the four cell types, respectively) (Fig. 3i).

### Endurance test of autofluorescence suppression

**Figure 4:**
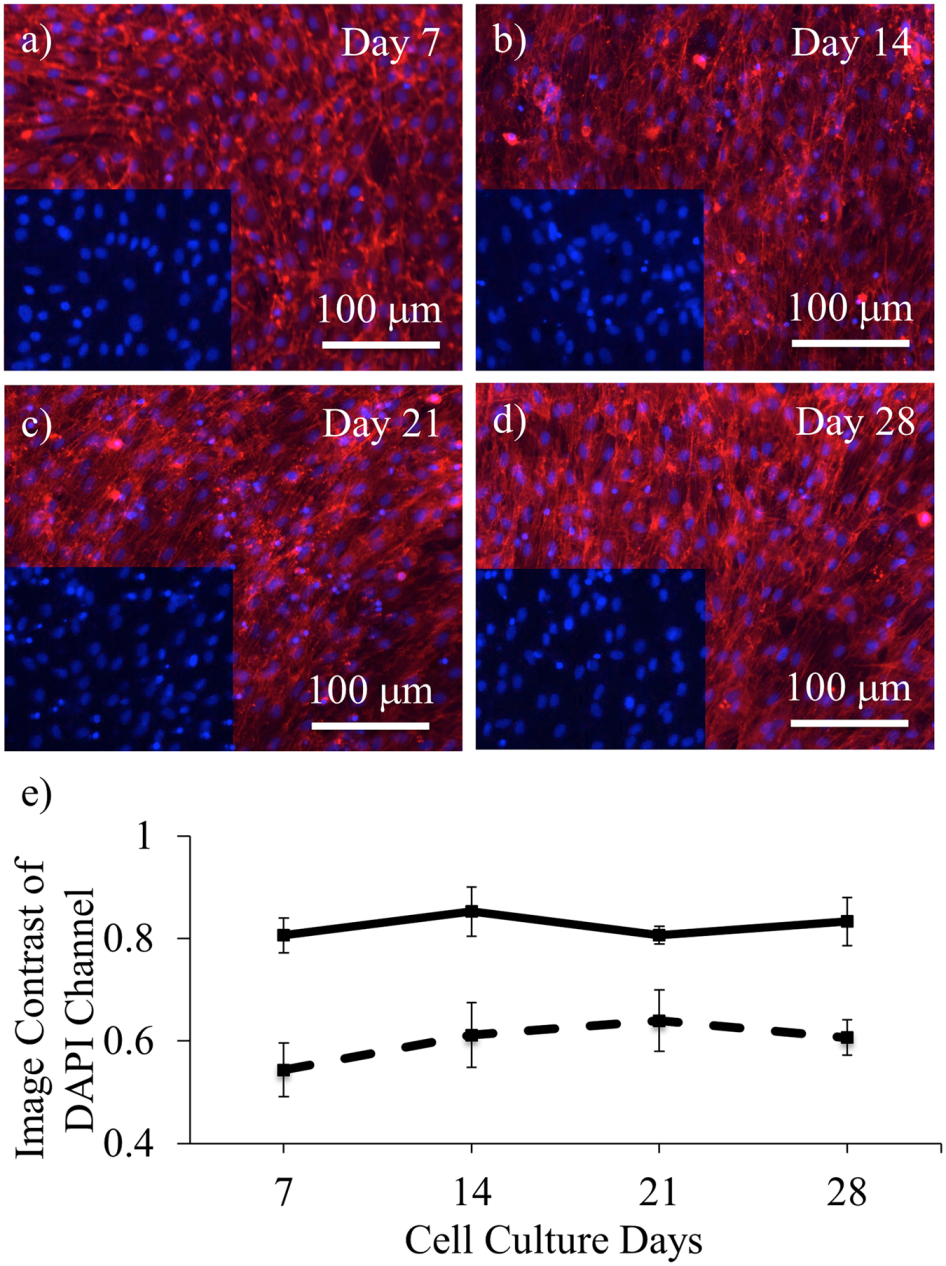
The autofluorescence suppression of pre-culture SBB treatments during long-term culturing. (**a**–**d**) Fluorescence images of F-actins and nuclei of 3T3 fibroblasts cultured for 28 days on PCL slabs with 0.3% pre-culture SBB treatments. (**e**) The image contrast comparison in DAPI channel. The SBB-treated samples are denoted by the solid line and non-treated samples by the dash line. For each group, there is no statistically significant change in the image contrast over time. For (**a**)–(**d**), the cells were stained by rhodamine-phalloidin and DAPI on Days 7, 14, 21, and 28 of cell culturing. Red denotes F-actins; and blue denotes cell nuclei.

The autofluorescence suppressive effect should preferably span over the entire period of cell culturing. In tissue engineering, most examinations of cell responses to the scaffolds are within the first few weeks of cell seeding^29–33^. During the period, polymer degradation may change the dimensions and molecular structures of the scaffolds^15, 34, 35^, and in turn affects the autofluorescence suppression capability of SBB treatments. 3T3 fibroblasts were cultured on PCL slabs. The autofluorescence suppression was examined on Days 7, 14, 21, and 28 (Fig. 4a–d). For the SBB-treated groups, the image contrast in DAPI channel did not show statistical changes over time (Fig. 4e). This suggested that SBB treatment has long-term autofluorescence suppressive capability. Here, the F-actins in TRITC channel were not used for calculating the image contrast due to the fact that the very high cell confluence after long-term culturing left too little uncovered area for image contrast determination.

### Effect of SBB treatment on cell behaviors

The effects of SBB treatments on cell behaviors were investigated using 3T3 fibroblasts and C2C12 skeletal myoblasts.

**Figure 5:**
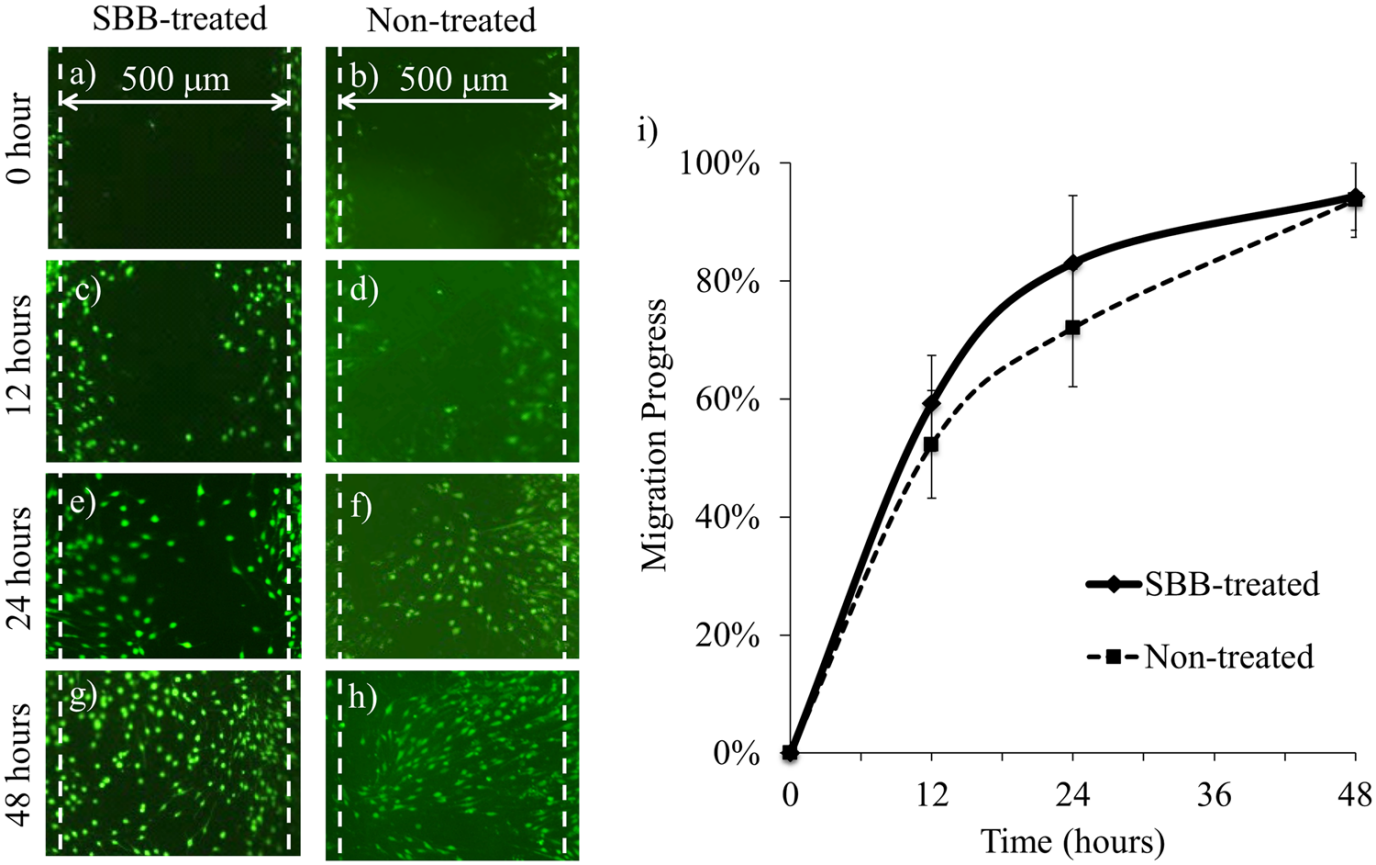
The effect of pre-culture SBB treatment on 3T3 fibroblast migration. (**a**–**h**) The fluorescence images of NIH/3T3 cells over time. (**i**) The change of 3T3 migration progress over time. At each time point, there was no statistical difference in the migration progress between the SBB-treated and non-treated groups. The width of initial scratch was ~500 μm. The cells were stained by calcein AM.

Fibroblast migration is important in wound healing and pathological fibrosis. The scratching experiment using 3T3 fibroblasts (Fig. 5a–h) showed that the cell migration progress was ~56% after 12 hours, ~78% after 24 hours, and ~100% after 48 hours on both the SBB-treated and non-treated surfaces (Fig. 5i). There was no statistical  difference in the cell migration progress between SBB-treated and non-treated groups at each time point, indicating that the SBB treatment does not significantly affect 3T3 migration capability.

**Figure 6:**
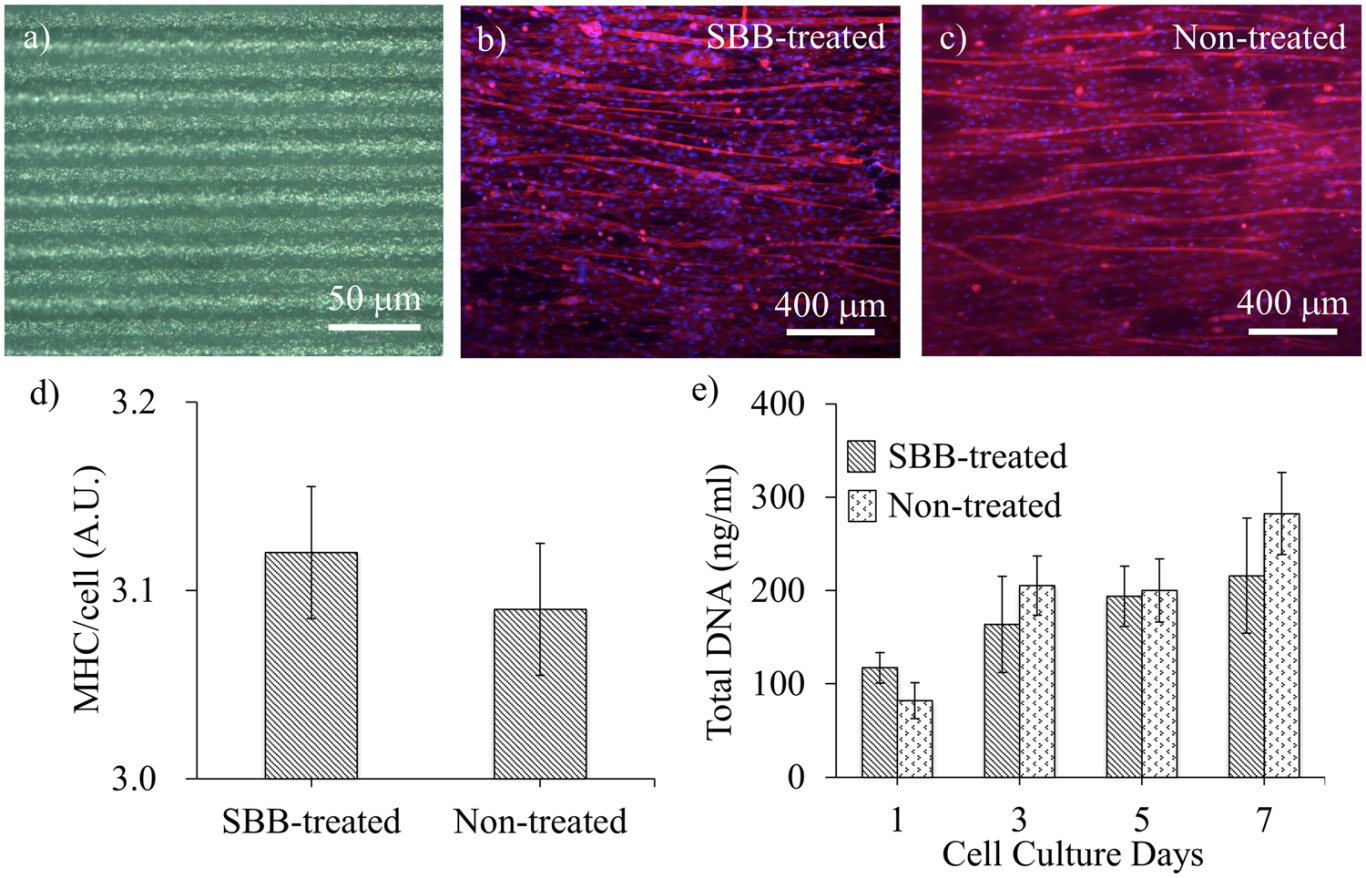
The effect of pre-culture SBB treatment on myogenic differentiation and cell proliferation for C2C12s. (**a**) The micrograph of a microgrooved PCL substrate with the period of 30 μm. (**b** and **c**) The fluorescence micrographs of SBB-treated and non-treated microgrooved PCL substrates after 7 days following the addition of the differentiation medium. The microgrooves were laid horizontally. (**d**) Comparison of the degree of myogenic differentiation with and without pre-culture SBB treatment. The degree of myogenic differentiation was determined by the expression of MHC (myosin heavy chain) per cell. (**e**) Cell proliferation was represented by the DNA amount of C2C12 cells cultured on the planar PCL slabs in the growth medium (for up to 7 days). On each day, there was no statistical difference in the total DNA amount between SBB-treated and non-treated groups. For (**b**,**c**), the myotubes were stained by rhodamine-phalloidin and DAPI on Day 7 following differentiation. Red denotes F-actins; and blue denotes nuclei.

Skeletal myoblasts have the capability of forming muscle tissues through myogenesis process. Periodic microgrooves were used as the contact guidance to facilitate linear myotube formation (Fig. 6a). After 7 days following the addition of the differentiation medium, most myotubes aligned along the longitudinal direction of the microgrooves in both the SBB-treated and non-treated groups (Fig. 6b and c). A large amount of long myotubes (>1 mm) was observed in both groups. ELISA analysis showed that the MHC expression/cell was not statistically different between the SBB-treated and non-treated groups (p = 0.2922), indicating that SBB treatment does not significantly affect the myogenic differentiation. The effect of SBB treatment on cell proliferation rate was examined by culturing C2C12 on planar PCL slabs and in the growth medium up to 7 days. The results showed that there was no statistical difference in DNA content between SBB-treated and non-treated groups of the same day (p = 0.0752 on Day 1, p = 0.3927 on Day 3, p = 0.8300 on Day 5, and p = 0.2018 on Day 7) (Fig. 6e).

### Image contrast improvement for cells cultured on 3D porous PCL scaffolds

**Figure 7:**
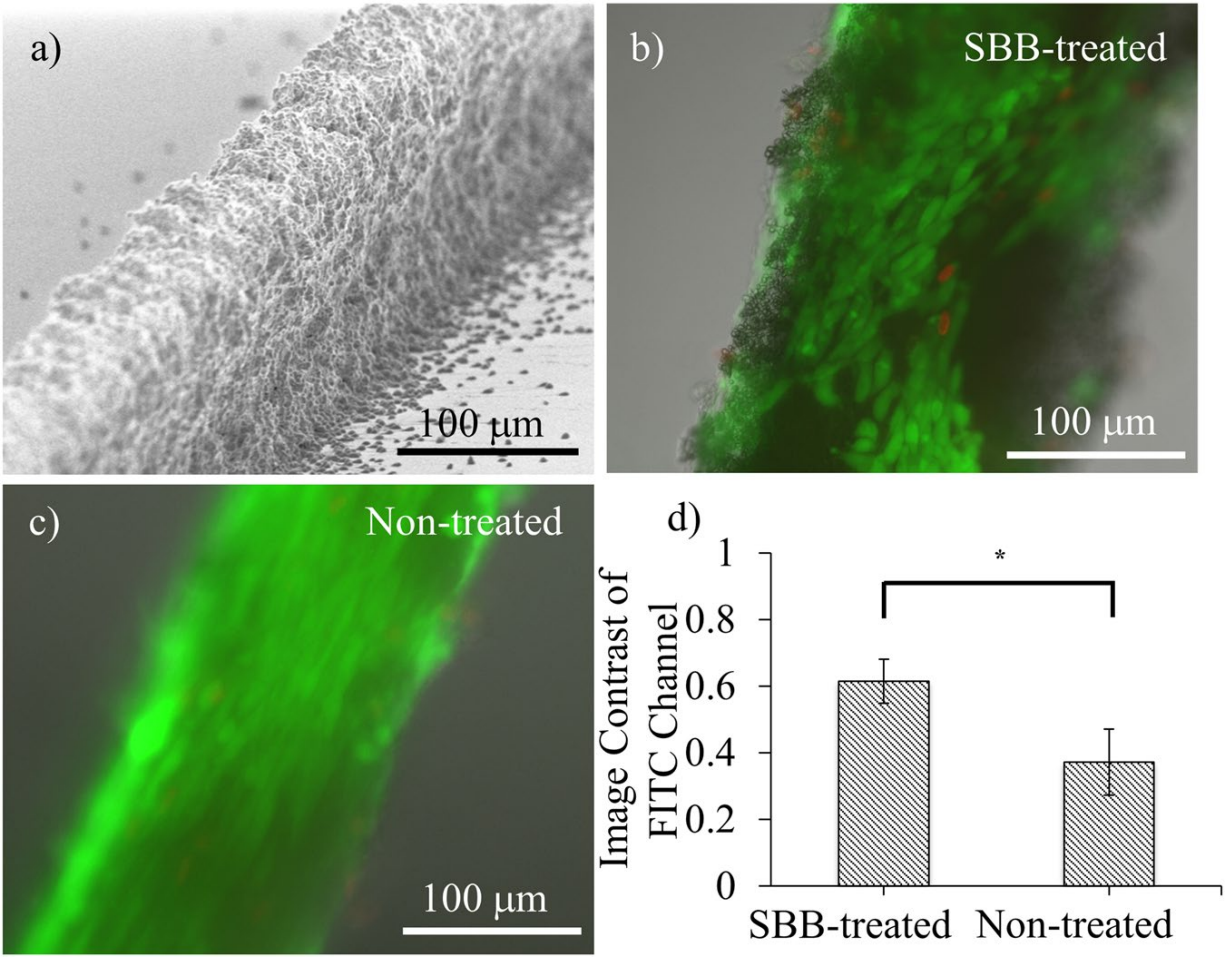
Autofluorescence suppression for cells cultured on 3D porous PCL scaffolds. (**a**) SEM micrograph of a representative 3D porous PCL scaffold; (**b** and **c**) The fluorescence images of C2C12 cultured on SBB-treated and non-treated PCL scaffolds. (**d**) The image contrast comparison. The cells were labeled by live/dead assay on Day 3 after cell seeding. Live cells are denoted by green. Dead cells are denoted by red.

PCL particulate structures were fabricated and served as 3D porous scaffolds. C2C12 myoblasts were cultured on the scaffolds, and stained by live/dead assay on Day 3. A representative SEM micrograph of such a scaffold is shown in Fig. 7a (~80 μm in width and ~100 μm in height). Similar to the results from planar PCL slabs, SBB-treated 3D porous PCL scaffolds (Fig. 7b) exhibited statistically lower autofluorescence signals than the non-treated groups (Fig. 7c) in FITC channel. Quantitative analysis showed that the image contrast decreased from 0.616 ± 0.066 in SBB-treated 3D scaffolds to 0.372 ± 0.100 in non-treated 3D porous scaffolds (Fig. 7d). The change was statistically significant (p = 0.0242).

### Comparison with pre- and post-permeabilization SBB treatments

**Figure 8:**
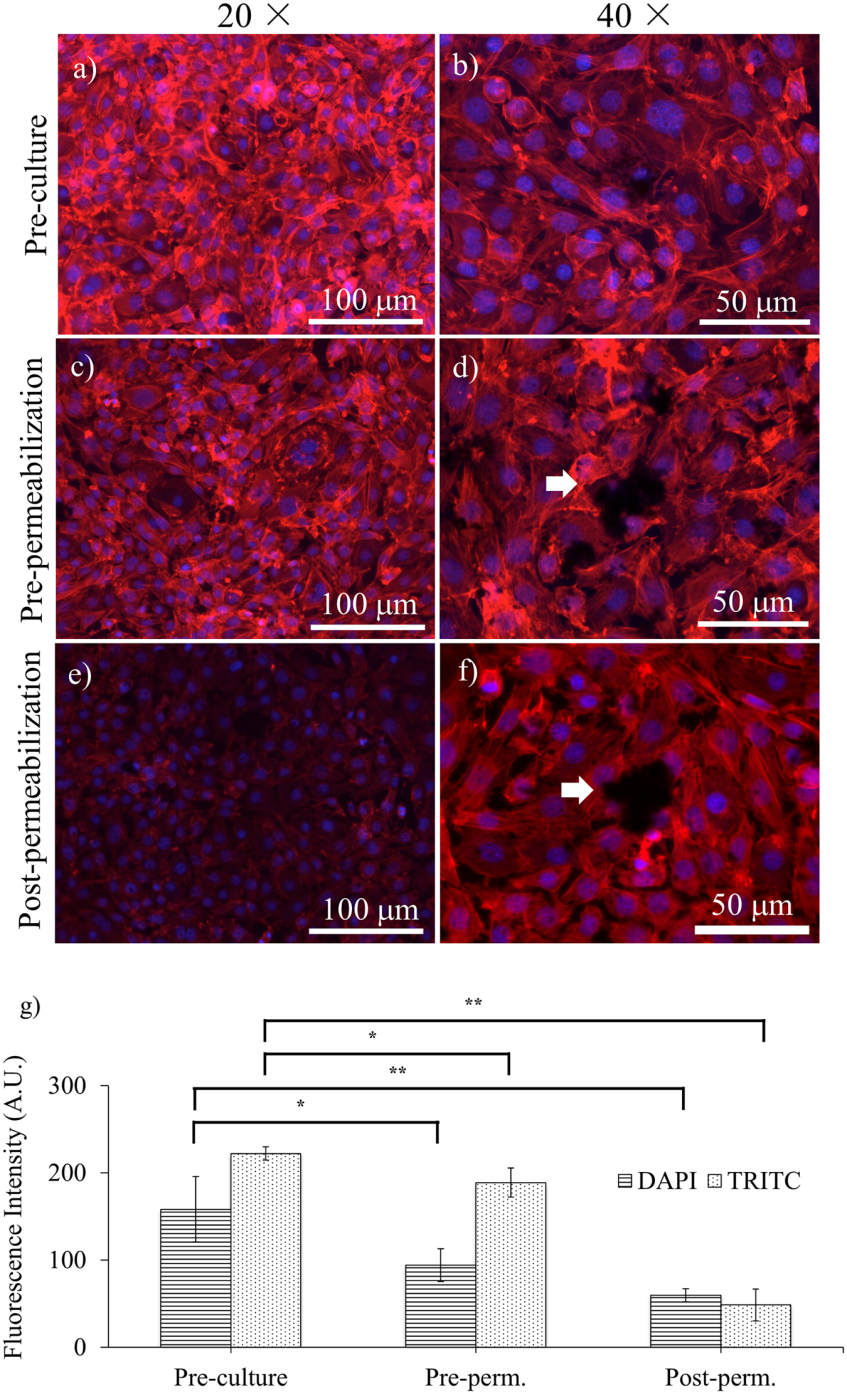
The comparison of three different SBB treatment schemes. The fluorescence images of C2C12 on planar PCL slabs after 3 days of cell culturing. The PCL slabs were treated by (**a**,**b**) pre-culture SBB treatment; (**c**,**d**) pre-permeabilization SBB treatment; and (**e**,**f**) post-permeabilization. The white arrows in (**d**) and (**f**) indicate dark particles on top of cells. The images in (**a**,**c**,**e**) were acquired with the same exposure time, analog gain, and lookup tables (LUTs) setting to ensure the fluorescence intensities in different images are comparable. The exposure time, analog gain, and lookup tables (LUTs) setting in (**b**,**d** and **f**) were adjusted to ensure the best brightness and contrast for analysis. (**g**) Statistical analysis of the fluorescence intensities of F-actins and cell nuclei obtained by the three treatment schemes. The cells were stained by rhodamine-phalloidin and DAPI on Day 3 of cell culturing with the growth medium. Red denotes F-actins; and blue denotes cell nuclei.

In our study, PCL scaffolds were treated with SBB prior to cell seeding, while the previous protocols treated the substrates with SBB after cell fixation, either prior to or after cell membrane permeabilization. To compare the impacts of different SBB treatment protocols on autofluorescence suppression, planar PCL slabs were treated using all the three protocols and cultured with C2C12 myoblasts. The fluorescence images acquired after 3 days culturing showed that all the protocols were able to suppress autofluorescence signals emitted from the PCL slabs in DAPI and TRITC channels (Fig. 8). The intensities of the fluorescence images in the pre-culture group were 158.171 ± 37.428 (DAPI) and 222.117 ± 7.749 (TRITC), comparing to 94.222 ± 18.877 (DAPI) and 188.867 ± 16.591 (TRITC) in the pre-permeabilization group and 59.567 ± 7.480 (DAPI) and 48.667 ± 18.180 (TRITC) in the post-permeabilization group. Statistical analysis showed that the pre-culture SBB treatments exhibited statistically higher intensity than the pre-permeabilization group (p = 0.0443 for DAPI and p = 0.0347 for TRITC) and the post-permeabilization group (p = 0.0071 for DAPI and p = 0.0013 for TRITC). In addition, a number of dark particles were observed in the pre-permeabilization and the post-permeabilization groups: some particles laid on the top of cell bodies and disrupted fluorescence images, as indicated by the arrows in Fig. 8d and f. Such particles were not observed in the pre-culture SBB group (Fig. 8b). The exposure time, analog gain, and lookup tables (LUTs) setting at 40× (Fig. 8b,d and f) were adjusted to obtain the best brightness and contrast.

## Discussion

In this study, PCL slabs were stained by SBB prior to cell seeding and culturing. The result showed that this pre-culture SBB treatment is capable of suppressing autofluorescence signals emitted from the PCL slabs and enhancing the contrast of fluorescence images. The cytotoxicity of the treatment was examined using live/dead assays with four cell lines and one group of primary cells. All the experiments showed that there was no statistical difference in the ratios of live/dead cells between the experimental and the control groups. Quantitative measurements of representative cell behaviors, including the migration progress of fibroblasts, and the myogenic differentiation and proliferation capability of skeletal myoblasts, confirmed that the pre-culture SBB treatment has little effects on changing cell behaviors. Pre-culture SBB treatment with significantly reduced background autofluorescence can thus provide a powerful tool for examining cell responses to the scaffolds in live cells with better image contrasts. The comparison of the mechanisms and performance of this treatment and of other SBB treatment scheme﻿s are as below.

### Mechanism of autofluorescence suppression by SBB

SBB has poor solubility in aqueous solution but soluble in several organic solvents. Due to the hydrophobic effect, SBB is more affinitive to hydrophobic surfaces^36, 37^. SBB staining is, therefore, more of a physical adsorption by the hydrophobic regions of the subjects, rather than bonding through chemical reactions. The autofluorescence suppressive effect of SBB is believed mainly attributed to its superior light absorption capability, which masks autofluorescence (*i*.*e*. absorbing autofluorescence and/or scattering light that is generated from histological samples^38–40^ and polymeric scaffolds^22^). This is different from some other fluorescence suppressive methods, such as photobleaching where the fluorophores are consumed through chemical reactions. Jaafar and colleagues examined the effect of the post-permeabilization SBB treatment for several polymers through ATR-FTIR and UV-vis spectroscopy. They concluded that SBB has minor chemical interactions with the polymers, but showed a significant light absorption for all examined polymers^21^. Although PCL was not tested, the result of PLCL, a copolymer of PCL and PLA (polylactic acid), is expected similar to that of PCL. Therefore, it is plausible to deduce that the mechanism underlying the image contrast improvement of the pre-culture SBB treatment is similar with post-culture SBB treatments: by absorption of autofluorescence signals emitted from the polymers.

### Comparison with other SBB treatment schemes

As shown in Fig. 8c and e, the fluorescence intensity of the post-permeabilization group was statistically lower than that of the pre-permeabilization group. This finding is in consistence with the results of previous reports^22, 38^. The fluorescence intensity reduction is believed due to the destruction of intracellular actin filaments by the organic solvent used to dissolve SBB. This makes post-permeabilization protocol less ideal for imaging cells with modest amount of cytoskeletons or low-abundance of proteins^41^. Similar fluorescence intensity reduction was found in pre-permeabilization SBB treatment protocols when ﻿comparing to pre-culture SBB protocols. This implied that intracellular structures may be destructed even without cell membrane permeabilization if the fixed cells are exposed long to organic solvents. Therefore, in pre- and post-permeabilization approaches, the SBB treatment duration should be minimized to reduce the destruction of intracellular structures. Unfortunately, the intensity reduction of autofluorescence signals emitted from polymer scaffolds increases with SBB treatment duration^22^. Such trade-off makes it challenging to determine the optimal SBB treatment duration in the pre- and post-permeabilization protocols. In this study, since SBB treatment is prior to cell culturing and fluorescence staining, the suppression of autofluorescence intensity of polymer scaffolds is independent to the fluorescence signal modulation for the cells. Long SBB treatment, which is especially useful for staining 3D porous or thick scaffolds^21^, can be used without sacrificing the image contrast.

The image quality of pre- and post-permeabilization groups is also affected by the dark particles as shown in Fig. 8d and f. These particles were believed caused by the solubility difference of SBB in ethanol (10 mg/ml) and water (0.1 mg/ml)^42^. After treating the polymer scaffolds with SBB solution (SBB in 70% of ethanol), aqueous solutions (e.g. phosphate buffer solution (PBS)) are used in the cell staining procedure. The excessive SBB dissolved in the ethanol solution may precipitate into aqueous solutions, which either deposits on top of cells and scaffolds or suspends in the aqueous solutions, deteriorating the fluorescence image quality. The pre-culture SBB treatment avoids this issue by removing excessive SBB prior to cell culturing. It also positions cells as the top-most layers, ensuring good image quality and contrast.

In summary, we reported an approach to treating polymeric scaffolds with SBB prior to cell seeding and culturing, in order to suppress the background autofluorescence of the scaffold materials while retaining the fluorescence signals emitted from the targets of interest. The assessments of cell viability, migration, proliferation, and myogenic differentiation show that this pre-culture SBB treatment protocol does not significantly affect cell behaviors. The autofluorescence suppression does not diminish during cell culture up to 28 days. The capability of this approach in enhancing the fluorescence intensity and the image contrast was experimentally validated using planar PCL slabs and 3D porous PCL scaffolds. Collectively, the results showed that this approach has a promising potential of improving fluorescence image quality of live cell-polymer scaffold complex, which may allow close examination of dynamic cell responses to external stimulations and to 3D extracellular environments.

## Methods

### Preparation of planar polymer slabs

PCL (M_n_ ~ 45,000; Sigma-Aldrich, MO, USA) was dissolved in acetone (Fisher Scientific, PA, USA) at a concentration of 10% (w/v), and molded by solvent casting. The polymer solution was dispensed into 2 cm^2^ cell culture plates and evaporated to cast a 3 mm thick slab. SBB solutions with the concentrations of 0.0005% to 1% (saturated) (w/v) were prepared by dissolving SBB powder (Sigma-Aldrich) in 70% (v/v) ethanol, and syringe-filtered (0.2 μm). The PCL slabs were then immersed in the SBB solution overnight at 4 °C. After staining, the PCL slabs were rinsed in 1× phosphate-buffered saline (PBS; Fisher Scientific) three times and exposed to UV light overnight for sterilization. Before cell seeding, all PCL slabs were coated with fibronectin (Sigma-Aldrich) at a concentration of 50 μg/ml overnight at 4 °C to enhance cell adhesion.

### Fabrication of grooved topographical PCL substrates

PCL slabs with microgrooved topography (linear microgrooves with the period of 30 μm and the amplitude of ~2.8 μm) were fabricated by solvent casting in a pre-molded polydimethylsiloxane (PDMS) template. The PDMS template was replica molded by dispensing and curing PDMS pre-polymer (Sylgard@184, Dow Corning, MI) on a pre-fabricated silicon wafer at 65 °C. The silicon wafer, with the grooved geometry with 30 μm in period, was fabricated by standard photolithography.

### Fabrication of three-dimensional (3D) PCL scaffolds

3D porous PCL scaffolds were fabricated using our previously described method^43^. Briefly, 10% (w/w) PCL/acetone solution was electrosprayed towards a collecting substrate that has been patterned with interdigitated microelectrodes. By connecting one group of microelectrodes at ground and keeping another group floated, PCL microparticles were attracted to the ground microstructures and accumulated into 3D particulate scaffolds. After detaching the scaffolds from the collecting substrate, the 3D porous structures were treated by SBB and fibronectin using the same procedure as for planar PCL slabs.

### Cell culturing

NIH/3T3 fibroblasts, C2C12 cells, PYS-2 endodermal cells (ATCC, VA, USA) were cultivated in Dulbecco’s modified Eagle’s medium (DMEM) with 10% fetal bovine serum and 1% penicillin/streptomycin (P/S) (all from Sigma-Aldrich). HUVECs (Lonza, MD, USA) were cultivated in Endothelial Basal Medium-2 (EBM-2) (Lonza) supplemented with growth factor kits. The four types of cells were seeded on planar PCL slabs at the density of 5 × 10^4^ cells/cm^2^. C2C12 cells were also seeded on grooved PCL substrates and 3D PCL scaffolds at the density of 5 × 10^4^ cells/cm^2^. The cells were then incubated at 37 °C with 5% CO_2_ to allow cell attachment and spreading. C2C12 differentiation was induced by changing to the differentiation medium (DMEM+2% horse serum+1% P/S) and keeping the cells in the medium for 7 days. For all the above culturing conditions, the media were changed every other day. The hippocampi of embryonic day 18 (E18) rat embryos were used to generate the primary neuron culture following an established protocol^44^. The dissociated primary cells (mostly hippocampal neurons with some glial cells) were cultured on the SBB-treated and non-treated PCL slabs at the seeding density of 5 × 10^4^ cells/cm^2^ with plating medium (DMEM+10% FBS+1% P/S, with 0.45% glucose, 1 mM sodium pyruvate, 25 mM glutamine, all from Thermo-Fisher). After 4 hours of plating, the medium was replaced by pre-warmed maintenance medium (neurobasal medium (Thermo-Fisher) with 2% of 50× B-27 supplement (Thermo-Fisher), 0.5 mM glutamine and 1% P/S). A half volume of the medium was changed with fresh maintenance medium every other day. All animal experiments were conducted in accordance with ethics guidelines stipulated by the Ohio State University animal ethics committee and with the NIH Animal Use Guidelines.

### Fluorescence labeling

The cytoplasm was labeled by incubating cells in PBS with 2 mM calcein AM (Life Technologies, CA) for 30 min. Afterward, the cells were fixed by incubation in PBS with 4% paraformaldehyde (Sigma-Aldrich) for 30 min at room temperature. The cell membranes were permeabilized by incubation with 0.1% Triton X-100 for 30 min. F-actins were stained by incubation with rhodamine-phalloidin (Life Technologies) for 30 min at room temperature (25 °C). The cell nuclei were labeled by incubation with 4′,6-diamidino-2-phenylindole (DAPI) (Sigma-Aldrich) for 30 min at room temperature.

### Live/dead cells assay

Cell viability was examined by labeling live and dead cells using Live/Dead^**®**^ kits (Life Technologies). Briefly, cells were incubated in PBS with ethidium homodimer-1 (EthD-1, 4 µM) and calcein AM (2 µM) for 30 min. Live cells were labeled with calcein AM (green) and dead cells were labeled with ethidium homodimer-1 (red).

### Cell migration assay

The migration progress of fibroblasts was evaluated using scratching assay^27^. NIH/3T3 fibroblasts were cultured on SBB-treated and non-treated PCL slabs at the seeding density of 5 × 10^4^ cells/cm^2^ and incubated for 3 days to allow cell confluence. On Day 3, the culture surface was scratched using a 200 μL pipette tip, resulting in a cell-free area with the width of ~500 μm. The cell migration was monitored by labeling the cells with calcein AM. The cell migration distance was determined by the distance of individual cells in the leading edges of the scratch. The migration progress was normalized by dividing the migration distance by the initial width of each scratch.

### Quantification of myogenic differentiation

After 7 days of myogenic differentiation, the expression of myosin heavy chain (MHC) was determined using a commercial mouse MHC ELISA kit (cat#MBS756241, MyBioSource, San Diago, CA). The expression of MHC was determined by measuring light absorbance by examined samples at 450 nm. The cell number of each sample was determined by total DNA assay as described in the section below. The myogenic differentiation of C2C12 was determined by the MHC expression per cell.

### Total DNA assay

The DNA content of C2C12 was quantified on Days 1, 3, 5, and 7 using a CyQUANT^®^ Cell Proliferation Assay kit (Thermo-Fisher, MA, USA). Briefly, cells were lysed in 200 μL of 1× cell lysis buffer and centrifuged to collect the supernatants containing DNAs. A standard curve of DNA content was obtained using serially diluted bacteriophage λDNA with the concentration ranging from 50 to 1000 ng/mL. All the DNA samples (50 μL) were then transferred into a 96-well plate. CyQuANT^®^ GR working solution (50 μL) was added to each well. After incubating the samples at room temperature for 5 min, the fluorescence intensity of the samples was measured using a fluorescence microplate reader (BioTek, VT, USA) with a filter with 485 nm excitation wavelength and 535 nm emission wavelength.

### Fluorescence imaging and analysis

Fluorescence microscopy was used to examine cells on planar PCL slabs and on 3D porous PCL scaffolds using Nikon fluorescence microscope Eclipse 80i with 4× to 40× objective lenses. Three fluorescence filters, namely DAPI (393 nm), FITC (483 nm), and TRITC (555 nm) were used. The intensities of the targets of interest and of the background were measured using Nikon NIS-Elements AR software. The image contrast (*D*) is calculated as:

1 $$D=\frac{{I}_{c}-{I}_{b}}{{I}_{c}+{I}_{b}}$$

where *I*_*c*_ and *I*_*b*_ denote the intensity of targets of interest (*e*.*g*. cytoplasm, F-actins, cell nuclei), and background fluorescence intensity, respectively. A high *D* indicates a high signal emitted from the targets of interest and a low background signal, and vice versa. To avoid overexposure, the intensity of the excitation light is modulated to achieve the best imaging quality of FITC channel, since it has the highest fluorescence intensity.

### Statistical analysis

Student *t*-test was used to examine whether there are statistical differences in the intensities of fluorescence images between different SBB concentrations; whether there are statistical differences in the image contrast of fluorescence images; the ratio of live/dead cells, the migration progress, MHC expression/cell, and the total DNA amount between SBB-treated and non-treated groups; and whether there are statistical differences in the intensities of fluorescence images between different SBB treatment protocols (pre-culture, pre-permeabilization, and post-permeabilization). For the image contrast and fluorescence intensity analyses, each group consists of four PCL samples (*i*.*e*. planar slabs or 3D porous scaffold). There are three images taken at the same magnification in each sample, resulting in a total of 12 images in each group. For the live/dead cells ratio and the migration progress evaluation, each group consists of three PCL slabs, and three images are acquired from each slab (total 9 samples). For MHC expression/cell and the total DNA amount evaluations, each group consisted of three PCL slabs, and two samples were collected from each PCL slab (total 6 samples). Statistical analysis was performed using JMP 11.0 (SAS^**®**^, NC, USA). ^(*)^p < 0.05 is considered significant and ^(**)^p < 0.01 is considered highly significant.
